# Comparison of Night, Day and 24 h Motor Activity Data for the Classification of Depressive Episodes

**DOI:** 10.3390/diagnostics10030162

**Published:** 2020-03-17

**Authors:** Julieta G. Rodríguez-Ruiz, Carlos E. Galván-Tejada, Laura A. Zanella-Calzada, José M. Celaya-Padilla, Jorge I. Galván-Tejada, Hamurabi Gamboa-Rosales, Huizilopoztli Luna-García, Rafael Magallanes-Quintanar, Manuel A. Soto-Murillo

**Affiliations:** 1 Unidad Académica de Ingeniería Eléctrica, Universidad Autónoma de Zacatecas, Jardín Juarez 147, Centro, Zacatecas 98000, Mexico; jr.ruiz68@uaz.edu.mx (J.G.R.-R.); hlugar@uaz.edu.mx (H.L.-G.); alejandro.somu@uaz.edu.mx (M.A.S.-M.); 2 LORIA, Université de Lorraine, Campus Scientifique BP 239, 54506 Nancy, France; 3 CONACYT, Universidad Autónoma de Zacatecas, Jardín Juarez 147, Centro, Zacatecas 98000, Mexico

**Keywords:** motor activity, depression, depressive episodes, data mining, random forest, night

## Abstract

Major Depression Disease has been increasing in the last few years, affecting around 7 percent of the world population, but nowadays techniques to diagnose it are outdated and inefficient. Motor activity data in the last decade is presented as a better way to diagnose, treat and monitor patients suffering from this illness, this is achieved through the use of machine learning algorithms. Disturbances in the circadian rhythm of mental illness patients increase the effectiveness of the data mining process. In this paper, a comparison of motor activity data from the night, day and full day is carried out through a data mining process using the Random Forest classifier to identified depressive and non-depressive episodes. Data from Depressjon dataset is split into three different subsets and 24 features in time and frequency domain are extracted to select the best model to be used in the classification of depression episodes. The results showed that the best dataset and model to realize the classification of depressive episodes is the night motor activity data with 99.37% of sensitivity and 99.91% of specificity.

## 1. Introduction

Wearable systems have been extensively used in healthcare field for several years. Physical medicine and rehabilitation were the first disciplines to venture into the implementation of these devices, in order to monitor the physical activity of the individual in pursuance of better disease diagnosis or patient ailment rehabilitation [1]. With the introduction of Internet of Things (IoT), wearable devices have been used to collect data from patients, not only for the detection of motor activity, but also for measuring blood pressure, heartbeat and even glucose level. This has allowed patients to be monitored any time and anywhere [2]. However, although the sensors are placed in the human body in the most normal and natural way possible to sense its activity, public data acquired from this type of sensing are still rare and not public [3].

Wearables have been used for the detection of depression according to their motor activity, since this represents a high indicator of the presence of this disease. Retardation or decrease in activities is the main feature for patients suffering from depression, for that reason, collect data from this condition could present good results that could be useful in different applications [4].

According to the World Health Organization (WHO) depression is the leading cause of disability [5], seven percent of people around the world suffer from Major Depressive Disorder (MDD), which causes the deterioration of the quality of life, the increase in medical costs and the death rate.

MDD is characterized by several syntopms as; loss of interest and pleasure in daily activities, sleep disorders, weight loss, suicide ideation, suicide attempts, among others. These symptoms must be present every day for at least two weeks to be diagnosed as depressive patients [6]. Depression is a treatable disease with a high level of efficacy using antidepressant medications and psychotherapy treatment, nevertheless for many patients being diagnosed can take months or even years to heal [7,8,9].

To diagnose or quantify the severity of the MDD, specialists use scales and manuals such as; the Hamilton Rating Scale for Depression written in 1960 [10], the Montgomery and Asberg Depression Rating Scale (MADRS) written in 1979 [11] or the Diagnostic and Statistical Manual of Mental Disorders (DSM). However, the use and interpretation of these methods depends largely on the ability of the specialist to determine the diagnosis [6,12,13,14]. A fact that discredits this type of methods is their lack of actualization and adaptation to the new advances and discoveries about the disease. In addition, these methods require the intervention of the patient, and in some cases the patients lie for any reason, causing the results not to be true and useful.

On the other hand, the use of data from monitoring depressive patients brings several benefits to medical services, mainly reduces the diagnosis and treatment time, improves the quality of life of patients and reduces medical costs [8].

Sensing motor activity arises as a favorable way for psychiatry and mental health to detect abnormal behaviors. Has been demonstrated that patients suffering from depression tend to reduce their daytime activity, and due to sleep disorders increase their nighttime activity [3]. In contrast, patients with bipolar disorder lead to an increase in their energy, however both scenarios presents a motor activity discrepancy from a healthy person. Therefore, circadian rhythm desynchronization is present in mental illness but is not well used for diagnosis or treatment monitoring yet [15].

The task of collect motor activity data can be accomplished using sensors like accelerometers, a combination of accelerometers, Global Positioning System (GPS), gyroscopes, inclinometers, magnetometers, etc. [1]. Nowadays, most of these technologies have very small dimensions, are cheep and easy to add in some specific devices or clothes, which facilitates usability and adaptability to everyday life. One way to replace these sensors could be using mobile phones, these devices have a big role in ubiquitous treatment, where the main idea is to avoid disturbances that the sensors or devices could generate on the patients and collect reliable data.

Once the data is collected the next step is to process it to recognize patterns and obtain some statistics or classifications. Sohrab Saeb et al [8] preset the relation of the regular clinical diagnosed and the sensor-based data from depression patients, they obtained an important result, in which a correlation between the GPS data and The Patient Health Questionnaire for depression (PHQ-9) scores was presented, this proves the relation between activity and depression [8].

In another work presented by Enrique Garcia-Ceja et al [3] a collected data from unipolar, bipolar and healthy control people was used to compare different machine learning algorithms and classify depressive and non-depressive signals, proving that through the use of machine learning techniques it is possible to classify between depressive and non-depressive people.

Machine learning is a set of algorithms that learn from the analyzed data to develop training models to classify that type of data. It allows among other applications, to make diagnoses or even predict some diseases [16]. These methods are commonly used in a data mining process that involves a series of steps related to each other and with the final objective of acquire valuable information.

Machine learning methods are increasingly used, EEG-based machine learning provides a non-invasive method to automatic diagnose MDD using algorithms like Linear Regression and Naive Bayes [17].

In this paper, a data mining process is carried out to classified depressive episodes using data collected during night time, day and full 24 h. The comparison between the classification using different data collected trough the time gives a better image of the disease and behavior of the patients with the diagnosis.

The structure of the paper is the Material and Methods, Results, Discussion and Conclusion sections, the Material and Methods section describes step by step the data mining process used to implement the classification of depressive and non-depressive episodes.

## 2. Materials and Methods

**Figure 1:**
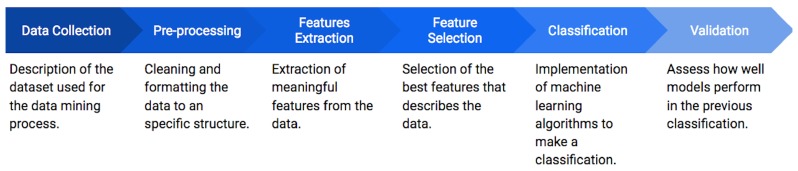
Data mining process used in this paper to classified depressive and non-depressive episodes.

The data mining process [18] shown in Figure 1 is followed to classify depressive episodes. The first stage consists on the data collection, where the Depresjon dataset containing the information of depression episodes from patients is acquired. These data are submitted to a pre-processing step in order to clean, normalize and segment them in one hour lapses. Then, a feature extraction is applied, where a set of 24 features in the time and frequency domain are obtained for different stages of the day (day, night and full day). A feature selection based on a forward selection (FS) approach is subsequently performed to reduce the number of features and to avoid redundant or non-significant information. From the selected features, a classification step based on the random forest (RF) algorithm is applied to develop a series of generalized models to identify between healthy and depressed patients according to the motor activity. Finally, these models are validated by a statistical analysis.

### 2.1. Dataset Description

In this work, the Depresjon dataset is used to classify depressive episodes. It is comprised by the motor activity of 23 patients diagnosed as bipolar, unipolar depressive and bipolar I (all these labeled as condition), and 32 non-depressive control subjects.

The motor activity corresponds to a weighting voltage collected with an actigraph watch (Actiwatch, Cambridge Neurotechnology Ltd., England, model AW4) located in the right wrist, which records movements over 0.5g in a sampling frequency of 32 Hz. Some advantages of actigraphs accelerometers is that they are inexpensive and well-known activity trackers [19] and, in addition, they are easy to wear and allow collecting data from day and night [20].

The data structure is formed by different files. One set of files contains a csv file per each condition and control with their recorded motor activity, organized in three columns: timestamp (one minute intervals), date (date of measurement), activity (activity measurement from the actigraph watch). Also, a scores file is included, which provides information about every subject. This file includes the columns: number (contributor id), days (number of days of data collection), gender (1 or 2 for female or male), age, afftype (1: bipolar II, 2: unipolar depressive, 3: bipolar I), melanch (1: melancholia, 2: no melancholia), inpatient (1: inpatient, 2: outpatient), edu (education), marriage (1: married or cohabiting, 2: single), work (1: working or studying, 2: unemployed/sick leave/pension), madrs1 (MADRS score when measurement started), madrs2 (MADRS when measurement stopped).

Features describing the date, timestamp and if it is or not a weekend, are not taking into account for the classification.

The package with the dataset files and full description of data can be downloaded from http://doi.org/10.5281/zenodo.1219550 [15].

### 2.2. Pre-Processing

For the pre-processing stage, the next step are proposed. Since the total amount of data recorded for each subject is different, a new subset of data is extracted, adjusting the number of observations to be equal for each subject. Theh, from the new set of data, a segmentation is applied to form one hour data intervals. This segmentation allowed the classification of depressive episodes per hour.

Therefore, based on the hourly segmentation, three different subsets are constructed; night motor activity (from 21 to 7 h taking into account the sunrise standard hours) [21], day motor activity (from 8 to 20 h) and finally all day motor activity with the total day hours. The number of observations contained in each dataset is shown in Table 1. After separated the data into day, night and 24 h data were cleaned from missing data.

Finally, the last step for the pre-processing is the cleaning of the data by the elimination of missing data, represented as NA, and the standardization of the motor activity. This standardization center the data into the mean mark, allowing to know how far the signal is from the mean point. This standardization is calculated by Equation (1).

(1) $$z_{i}=\frac{x_{i}-\bar{x}}{s},$$

where $x_{i}$ is the actual point of the activity data, $\bar{x}$ is the mean of the total motor activity data and *s* is the standard deviation of the total motor activity data.

### 2.3. Feature Extraction

For each dataset, the 24 features shown in Table 2 are extracted. This process is based on similar works that extract features from an accelerometer signal [22,23,24].

From the total features extracted, ten are based on the time-domain, as shown in Table 2, referred to the data collected by the actigraph every minute.

To transform the time-domain data into frequency-domain, the fast Fourier transform (FFT) is applied, which can be calculated with Equation (2),

(2) $$x\left(k\right)=\sum_{n=0}^{N-1}x\left(n\right)*e^{-j2\pi (x\frac{n}{N})},$$

where x(n) represents each motor activity collected per minute on an hour, *N* represents the total observations on an hour lapse, *k* represents the current frequency taking values from 0 to N−1, and x(k) represents the spectral components of the samples.

For this FFT process, the representation of the original signal in the frequency domain is computed using the discrete Fourier transformation (DFT). This representation is formed by complex numbers, eliminating the imaginary part of each number in the frequency-domain signal. For this transformation, it is needed to calculate the power spectral density (PSD), as shown in Equation (3),

(3) $$P=\lim_{T\to \infty }\frac{1}{T}\int_{0}^{T}{\left|x(k)\right|}^{2}dt,$$

where *P* represents the energy from the signal, *T* represents the length of the signal lapse and x(k) represents the frequency-domain signal. The spectrum is normalized by the length of the signal.

The 14 remaining features are extracted from the PSD of the signal to obtain the best characterization of the signal.

### 2.4. Feature Selection

The next step consists on reducing the dimension of the feature sets and selecting the best model for the description of the data. To accomplish the task, a FS approach is applied to the three sets of features (day, night and full day), using 70% of the data for the training of the model and the 30% remaining data for the testing of the model [25].

FS is implemented using the logistic regression (LR) classifier, since the nature of the data is binary (depressive, “1”, and not depressive, “0”, episode). Therefore, LR is used to model the selected features by FS for the classification of depressive episodes. For simplicity, each feature is labeled with a number, as shown in Table 3.

### 2.5. Classification

For the classification stage, the RF algorithm is used to classify depressive and non-depressive episodes based on the features selected for each dataset, specifically using the best ranked features according to the previous step.

According to Phan Thanh Noi et al [26], the RF algorithm use has been increasing in the past few years because of its effectiveness. RF algorithm came to light in 2001 created by Leo Breiman et al [27], it is conformed by a combination of trees generated randomly and with different predictors each of them. This algorithm is a supervised technique where multiple decision trees are used to develop a forest. This forest is more robust if it is developed with more number of trees for the classification.

To classify an observation, the trees are generated in order to response questions with yes/no response, every tree bases the response in the features of the observation and responded to make a classification of the observation [28].

Generally, a leaf is used for the expansion of the construction of the tree in each step. At the end, from the decision trees built, they are merged into a single tree to obtain a higher prediction accuracy [29].

The general performance of RF follows the next steps,

- Given a dataset $M_{1}$, of size m×n, a new dataset $A_{2}$ is created from the original data, sampling and eliminating a third part of the row data.
- The model is trained generating a new dataset through the reduced samples, estimating the unbiased error.
- At each node point (which are the points where the trees are growing simultaneously), the column $n_{1}$ is selected from the total *n* columns.
- When the trees finish growing, a final prediction based on the individual decisions is calculated, looking for the best classification accuracy.

For the implementation of the RF algorithm, it is used the R language [30] with the default settings of the randomForest library [31].

### 2.6. Validation

Finally, to evaluate the effectiveness of the classification process, a statistical validation is applied, based on nine metrics: true positive (TP) (conditions correctly classified), true negative (TN) (controls correctly classified), false positive (FP) (controls incorrectly classified), false negative (FN) (conditions incorrectly classified), sensitivity, specificity, positive predictive value (PPV), negative predictive value (NPV) and accuracy.

Sensitivity can be calculated by Equation (4),

(4) $$Sensitivity=\frac{TP}{TP+FN},$$

describing the true positive rate, i.e. the probability that a depressive episode is classified rightly.

Specificity can be calculated by Equation (5),

(5) $$Specificity=\frac{TN}{FN+TP},$$

describing the true negative rate, i.e. the probability that a non-depressive episode is classified rightly.

The PPV value can be defined by Equation (6), being the probability that a new episode of a person suffering from depression is classified as a depressive episode

(6) $$PPV=\frac{Sensitivity*Prevalence}{Sensitivity*Prevalence+(1-Specificity)*(1-Prevalence)},$$

where Prevalence is the percentage of observations with a condition, in this case depressive episodes.

The NPV value can be defined by Equation (7), being the probability of a episode with absence of depression is classified as negative.

(7) $$NPV=\frac{Specificity*(1-Prevalence)}{\left(1-Sensitivity)*Prevalence+Specificity*(1-Prevalence\right)},$$

Finally, the accuracy can be calculated with Equation (8), being the total probability that one episode is classified correctly.

(8) $$Accuracy=\frac{TruePositive+TrueNegative}{TruePositive+TrueNegative+FalsePositive+FalseNegative},$$

## 3. Experiments and Results

**Figure 2:**
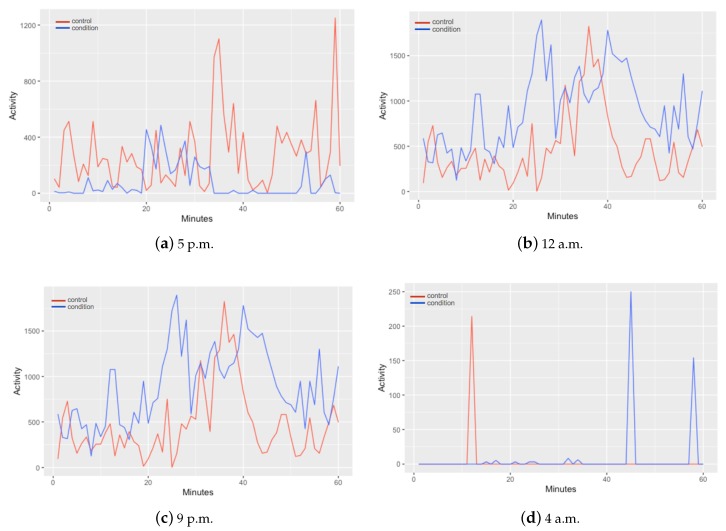
Comparison of motor activity in different hours of the day between a control an a condition.

In Figure 2 is presented a comparison of the motor activity between a control and a condition in different hours of the day. In the differences can be observed that every hour activity of control and condition shows different patterns. From these data, a segmentation is applied to form data intervals containing the information of one hour time lapses. The structure of the data for every observation is contained by 61 columns; one column for the monitored hour and one column for each minute (60 columns) of motor activity. This segmentation allowed the classification of depressive episodes per hour.

From Figure 2 they can also be distinguished different patterns on the activity of control and condition subjects in different moments of the day. In Figure 2a, at 5 p.m., the control subject signal collected presents higher levels of activity in contrast with the depressive patient. This can be the most expected conduct of a patient with depression. Nevertheless, as the day ends the signal of both, control and condition, starts to change as shown in Figure 2b–d, depressive activity containing higher values.

Therefore, based on this, the data is treated in three different sets, each one corresponding to different moments of the day. One set corresponding the day, one to the night and one to the full day.

Then, for the next stage a feature selection is proposed. The results for each dataset are shown in Table 4. Accuracy is the metric used to evaluate the performance of the models constructed by the FS approach including different number of features (two, four, five, six, seven, eight, nine and ten).

In case of Night Data and Full Day Data datasets, higher accuracy is achieved with nine-features model in classification of depressive and non-depressive episodes . Day data best model is comprised by 8 features, however, the difference with nine-features model is less than 0.1 percent.

After the feature selection with FS approach, validation step is done in two steps. Firstly, classification is done with the best nine-features model for each dataset, mentioned in Table 5, even when Day Data has higher accuracy with 8 features, best nine features is selected to compare the performance in same circunstances with the other two datasets. The results of this classification are shown in Table 6, described as Best 9 Features Day, Best 9 Features Full Day and Best 9 Features Night. In addition to accuracy, which was used in FS step, sensitivity and specificity were calculated in this validation to give a wide view of the performance of the models.

Secondly, classification is performed using the nine-features set of the Best 9 Features Night, applied to the Day Data and the Full Day Data datasets, because this nine features model is the one which outperforms all other nine features models in all proposed metrics. This in order to evaluate the performance of a general model, i.e. a unique model for all the time of the day. The results of this classification are described as the Best Model Day, Best Model Full Day in Table 6.

From this table can be observed that every model has a significant performance in the classification of depressive episodes. Sensitivity values oscillate from 98.24% to 99.37% and specificity range oscillates between 98.08% and 99.31%, establishing an almost perfect classification of depressive and non-depressive episodes.

The lowest, but still being good results, are those from the Day Data with nine features selected from the Night Data.

## 4. Discussion

In this section the discussion of the results obtained for the different stages applied in this work is presented. Initially, the features extracted from the data are submitted to a feature selection to remove redundant or non-significant features, preserving those that contribute most to the description of depressive subjects in the different moments of the day. Then, a classification is carried out, modeling the set of features that presented the best result in the previous step for the different moments of the day. A final validation is applied to statistically evaluate the performance of the models obtained.

According to the validation values shown in the previous section, the feature selection and classification stages allowed to obtain statistically significant results.

From the feature selection step, a series of feature sets have been obtained along with their calculated accuracies, shown in Table 4. The main objective in this step is to be able to select the smallest set of features obtaining the best accuracy. As can be seen, the accuracy follows an increase pattern for most cases each time a feature is increased in the set. However, for the three data sets it is observed that the accuracy stops increasing when the tenth feature is added, and even decreases for the Day Data and Full Day Data sets. For the Night Data and the Full Day Data, the best accuracy is calculated with the set of nine features, obtaining 0.7818 and 0.7792, respectively, while for the Day Data the best accuracy is calculated with the set of eight features, obtaining 0.7744. And, in general, of all the feature sets selected for the three data sets, the best accuracy is obtained with the nine-features set of the Night Data. Based on this and in order to make a direct comparison, the sets of nine features have been selected as the best for each data set. The description of the features included in these sets are shown in Table 5.

Comparing the best nine-features sets shown in Table 5, it can be observed that only for the Night Data the maximum (time) value is selected in the FS. This may be due to sleep disorders that make patients with depression more active at night, being possible to differentiate the level of motor activity of a person who does not have this condition, since it is regularly lower.

Another important detail shown in the description of the feature sets is the frequency-related features, since for the three different data sets the same features were selected in this domain. This demonstrates the robustness and generalization in the information provided by these features, since regardless of the time of day, it is possible to identify subjects with depression presence with the levels of activity that occur.

The next step corresponds to the modeling of the set of features selected for each dataset for a classification task, based on the RF technique. For this purpose, the nine-features sets selected for each dataset were submitted to the modeling. In addition, taking into account that of all the selected feature sets, the one with the best accuracy was the nine-feature set of the Night Data, another classification was made in the Full Day Data and Day Data using this nine-feature set. To avoid confusion, this classification is labeled as Best Model Full Day and Best Model Day, for the Full Day Data and the Day Data respectively.

It is important to mention that RF was selected for the classification since it has been used to classify the motor activity of depressed subjects in other works. Zanella-Calzada et al. [22] present the classification of depressive and no depressive episodes using RF, obtaining an accuracy of 0.893, while M. Pal et al. [32] compare the performance between RF and SVM, resulting RF more efficient even with fewer parameters to make the classification.

To measure the significance of the classification, the TP, TN, FP, FN, sensitivity, specificity, PPV, NPV and accuracy metrics were measured, obtaining the results shown in Table 6. Initially, it can be seen that the FP and FN values are not significant if they are compared with the TP and TN values, taking into account that the TP and TN are the conditions and controls, respectively, correctly classified being much higher than the FP and FN values, which are the subjects incorrectly classified. An important point to note is that the lowest number of FP and FN is obtained when the classification is carried out using the set of nine features selected for Night Data applied to the three different data sets, as can be seen in the Best Model Day and Best Model Full Day. However, the lowest number of FP and FN are obtained in the Night Data set, allowing to demonstrate that the data of these nine features, specifically in this period of the day, generate values in the levels of the motor activity that allow to identify the depressive subjects, reducing the ambiguities that may be obtained in the activity levels presented by the Day Data and Full Day Data sets.

For the rest of the results it can be seen that the highest values were obtained using the Night Day feature set, since if a comparison is made of the accuracy obtained from the classification of the nine features selected from the Data Day set and the accuracy obtained from this same data set but using the nine features of the Night Data (Best Model), an increase of 0.45% can be observed. In the case of Full Day Data it is observed the same behaviour, obtaining an increase in the accuracy of 0.43% when using the set of nine features of the Night Data. For the Night Day data, the classification accuracy is almost perfect, obtaining a value of 99.72%.

Based on this, it can be noted the great contribution generated by the set of features selected for the Night Data set, but specifically with the maximum (time) feature, since it is the main difference between this set of selected features and the others two sets, contributing to have significant behavior not only in the Night Data set, but in all sets. The maximum (time) feature represents the highest value obtained from activity level and specifically at night, it allows to identify depressive subjects almost perfectly. This may be because, according to Armitage et al. [33], around 80% of patients diagnosed with MDD suffer from sleep disorders. This represents an important change in the circadian rhythm of patients suffering from depression and a healthy persons, causing depressive subjects to have more motor activity. In Figure 2 can be notice that even in a sleepy hour for both control and condition (4 a.m.) patient suffering from MDD have more disturbances than the control subject.

Finally, it should be noted that, as mentioned above, the best results are obtained using the Night Data dataset with the set of nine features specifically selected for this dataset. While the lowest results are obtained using the Day Data data set, however, these values increase if the nine features selected for Night Data are used. For the Full Day Data set, an intermediate value can be observed between the other two datasets. Therefore, based on this, it can be known that the ambiguities in the classification of depressive subjects are greater during the day than during the night. This may be due to the fact that during the day people regularly must carry out daily activities, such as work or studies, regardless of whether they suffer from depression. While at night, the presence of this condition may be more evident due to the irregularities that it can cause to sleep, while people who do not suffer from depression can have a quieter sleep and therefore much less activity levels.

## 5. Conclusions

The main objective of this work is to develop a set of models that allows to classify depressive and not depressive episodes in different moments of the day (day, night and full day) based on the motor activity levels of subjects. For this purpose, a series of stages are applied to the Depresjon database, which describes the activity levels of patients with presence of depression and controls.

For the feature selection stage, it is used the FS technique based on LR, in order to obtain the set of features that provide the most relevant information for data modeling, while for the classification stage, it is applied the RF technique. For the validation of the performance of these steps, a series of statistical metrics are measured.

According to the results obtained, it can be observed that from the feature selection, the best set of selected features is obtained from the data that corresponds to the night period, since the best accuracy is calculated when classifying the subjects with these features using the activity levels presented during the night. This set is contained by nine features, being maximum (time) the feature that generates the greatest contribution, since it provides the maximum values of activity level during the night, which are generally related to the subjects that present depression.

Therefore, this allows us to conclude that it is possible to identify subjects with the presence of depression based on the model developed in this work using the data of motor activity levels. In addition, for the identification of this condition, it is sufficient for patients to only measure their activity levels through the actigraph during the night, since with these data, classification can be made through the model obtained allowing to know if the subject presents depression or not, with an accuracy of 99.72%.

It should be noted that this is a preliminary tool that can be of great support for specialists in the diagnosis of depression based on a non-invasive method, since it would only be necessary to have the patient’s activity level data to make the diagnosis at through this model.

## Figures and Tables

**Table 1:** Datasets created from Depresjon dataset.

Dataset	Observations
Day	14168
Night	11945
Full Day	26113

**Table 2:** Features extracted for the day, night and full day datasets, in time and frequency domain.

Feature	Equation	Time Domain	Frequency Domain
Mean (μ)	$1/n\sum_{i=1}^{n}x_{i}$ ^1^	•	•
Median	$x_{(n+1)/2}$	•	•
Standard deviation (SD, σ)	$\surd (\sum_{i=1}^{n}{\left(x_{1}-\bar{x}\right)}^{2}/\left(n-1\right))$	•	•
Variance	$1/n\sum_{i=1}^{n}{\left(x_{i}-\mu \right)}^{2}$	•	•
Kurtosis	$\mu_{4}/\sigma_{4}$ ^2^	•	•
Coefficient of Variance	σ/μ	•	•
Interquartile range	$Q_{3}-Q_{1}$ ^3^	•	•
Minimum	(Maximumvalue)	•	•
Maximum	(Minimumvalue)	•	•
Trimmed Mean	(truncatedmean)	•	•
Spectral Density	(defined above)		•
Entropy	$-\sum_{i}p\left(x_{i}\right)log_{2}p\left(x_{i}\right)$ ^4^		•
Skewness	$\bar{\mu_{3}}$ ^5^		•
Spectral Flatness	$(exp\left(1/N\sum_{n=0}^{N-1}lnx\left(n\right)\right)/(1/N\sum_{n=0}^{N-1}x\left(n\right)$ ^6^		•

${}^{1}$—*n* = total number of samples, $x_{i}$ = actual sample. ${}^{2}$—$\mu_{4}$ = μ of the fourth moment, $\sigma_{4}$ = SD of the fourth moment. ${}^{3}$—$Q_{3}$ = third quartile three, $Q_{1}$ = first quartile. ${}^{4}$—$p_{i}\left(x_{i}\right)$ = probability of $x_{i}$. It represents the media uncertainty of a random variable. ${}^{5}$—$\bar{\mu_{3}}$ = third standardized moment. ${}^{6}$—x(n) = magnitude of bin number *n*.

**Table 3:** Features extracted from the hourly motor activity segments.

Number	Time-Domain Feature	Number	Frequency-Domain Feature
**0**	Kurtosis	**10**	Kurtosis
**1**	Mean	**11**	Mean
**2**	Median	**12**	Median
**3**	SD	**13**	SD
**4**	Variance	**14**	Variance
**5**	Coefficient of variance	**15**	Coefficient of variance
**6**	Interquartil rank	**16**	Spectral density
**7**	Minimum	**17**	Interquartile rank
**8**	Maximum	**18**	Trim mean
**9**	Trim mean	**19**	Minimum
		**20**	Maximum
		**21**	Entropy
		**22**	Skewness
		**23**	Spectral flatness

**Table 4:** Forward Selection results for the night, day and full day data.

Night Data		Day Data		Full Day Data	
**Features**	**Accuracy**	**Features**	**Accuracy**	**Features**	**Accuracy**
[2,13]	0.7398	[2,13]	0.7525	[2,13]	0.7393
[2, 12, 13]	0.7683	[2, 12, 13]	0.7589	[2,12,13]	0.7623
[2,12,13,23]	0.7706	[0,2,12,13]	0.7628	[0,2,12,13]	0.7655
[2,8,12,13,23]	0.7763	[2,8,11,22,23]	0.7728	[0,2,12,13,15]	0.7725
[2,6,8,12,13,23]	0.7776	[0,2,9,12,13,23]	0.7728	[0,2,9,12,13,15]	0.7747
[2,6,8,12,13,15,23]	0.7792	[1,2,7,8,11,22,23]	0.7728	[0,2,9,12,13,15]	0.7747
[0,2,6,8,12,13,15,23]	0.7811	[1,2,5,7,8,11,22,23]	0.7744	[0,2,5,9,12,13,15,23]	0.7758
[0,2,6,7,8,12,13,15,23]	0.7818	[0,1,2,7,9,12,13,15,23]	0.7735	[0,2,5,7,9,12,13,15,23]	0.7792
[0,2,6,7,8,9,12,13,15,23]	0.7818	[0,1,2,5,7,9,12,13,15,23]	0.7731	[0,2,5,6,7,9,12,13,15,23]	0.7761

**Table 5:** Best nine features model in every dataset; day, night and full day.

Dataset	Best Nine Features
	kurtosis (time), mean (time), median (time), minimum (time),
Day	trim mean (time), median (frequency), SD (frequency),
	coefficient of variance (frequency), spectral flatness (frequency)
	kurtosis (time), median (time), interquartil rank (time),
Night	minimum (time), maximum (time), median (frequency), SD (frequency),
	coefficient of variance (frequency), spectral flatness (frequency)
	kurtosis (time), median (time), coefficient of variance (time),
Full Day	minimum (time), trim mean (time), median (frequency), SD (frequency),
	coefficient of variance (frequency), spectral flatness (frequency)

**Table 6:** RF results for datasets using the nine features models and the best model from the forward selection on night dataset.

Dataset	TP	TN	FP	FN	Sensitivity	Specificity	PPV	NPV	Accuracy
Best 9 Features Day	1455	2729	40	26	98.24%	98.56%	97.32%	99.06%	98.45%
Best Model Day	1470	2736	25	19	98.72%	99.09%	98.33%	99.31%	98.96%
Best 9 Features Full Day	2703	5047	53	31	98.87%	98.96%	98.08%	99.39%	98.93%
Best Model Full Day	2737	5047	19	31	98.88%	99.62%	99.31%	99.39%	99.36%
Best 9 Features Night	1259	2315	2	8	99.37%	99.91%	99.84%	99.66%	99.72%
